# Cyp26b1 Regulates Retinoic Acid-Dependent Signals in T Cells and Its Expression Is Inhibited by Transforming Growth Factor-β

**DOI:** 10.1371/journal.pone.0016089

**Published:** 2011-01-07

**Authors:** Hajime Takeuchi, Aya Yokota, Yoshiharu Ohoka, Makoto Iwata

**Affiliations:** 1 Laboratory of Biodefense Research, Faculty of Pharmaceutical Sciences at Kagawa Campus, Tokushima Bunri University, Sanuki, Japan; 2 Japan Science and Technology Agency, CREST, Tokyo, Japan; Charité-University Medicine Berlin, Germany

## Abstract

**Background:**

The vitamin A metabolite, retinoic acid (RA), plays important roles in the regulation of lymphocyte properties. Dendritic cells in gut-related lymphoid organs can produce RA, thereby imprinting gut-homing specificity on T cells and enhancing transforming growth factor (TGF)-β-dependent induction of Foxp3^+^ regulatory T cells upon antigen presentation. In general, RA concentrations in cells and tissues are regulated by its degradation as well. However, it remained unclear if T cells could actively catabolize RA.

**Methodology/Principal Findings:**

We assessed the expression of known RA-catabolizing enzymes in T cells from mouse lymphoid tissues. Antigen-experienced CD44^+^ T cells in gut-related lymphoid organs selectively expressed *Cyp26b1*, a member of the cytochrome P450 family 26. However, T cells in the spleen or skin-draining lymph nodes did not significantly express *Cyp26b1*. Accordingly, physiological levels of RA (1–10 nM) could induce *Cyp26b1* expression in naïve T cells upon activation *in vitro*, but could not do so in the presence of TGF-β. Overexpression of *Cyp26b1* significantly suppressed the RA effect to induce expression of the gut-homing receptor CCR9 on T cells. On the other hand, knocking down *Cyp26b1* gene expression with small interfering RNA or inhibiting CYP26 enzymatic activity led to enhancement of the RA-induced CCR9 expression.

**Conclusions/Significance:**

Our data demonstrate a role for CYP26B1 in regulating RA-dependent signals in activated T cells but not during TGF-β-dependent differentiation to Foxp3^+^ regulatory T cells. Aberrant expression of CYP26B1 may disturb T cell trafficking and differentiation in the gut and its related lymphoid organs.

## Introduction

The vitamin A metabolite, retinoic acid (RA), plays critical roles in many life processes including immune responses. Among the roles, the regulation of lymphocyte trafficking is essential for the gut immunity [1], [2]. We have previously found that RA induces the expression of the gut-homing receptors, α4β7 integrin and chemokine receptor CCR9, on T and B cells upon activation, and imprints them with gut-homing specificity [1], [3]. Activation of naïve T cells is dependent on antigen presentation by dendritic cells (DCs) in lymphoid tissues. DCs in gut-related lymphoid organs can produce RA by expressing the key RA-synthesizing enzyme, retinaldehyde dehydrogenase (RALDH), and imprint gut tropism on T cells during antigen presentation [1], [4], [5]. It has been also found that these DCs enhance the transforming growth factor (TGF)- β-dependent differentiation of naïve CD4^+^ T cells to Foxp3^+^ inducible regulatory T cells (iTreg) and suppress the TGF-β/IL-6-dependent differentiation of proinflammatory Th17 cells [6]–[11]. However, it remains unclear how RA is catabolized in T cells to prevent excessive RA stimulation.

It is known that RA-mediated signaling is regulated during the embryonic morphogenesis through coordinated regulation of RA synthesis and catabolism [12]. Several cytochrome P450 (CYP) enzymes are known to catabolize RA via several routes leading to a variety of polar catabolites; the immediate products include 4-hydroxy-RA, 4-oxo-RA, 18-hydroxy-RA and 5,6-epoxy-RA [13]. The predominant pathway is oxidation at the 4-position of the cyclohexenyl ring to form 4-hydroxy-RA [14]. Among the CYP enzymes, the CYP26 family (CYP26A1, CYP26B1 and CYP26C1) is likely to be responsible for much of the RA-inducible RA metabolism. Each *Cyp26* gene has a distinct pattern of organ-specific expression during early embryogenesis [15]–[17]. RA levels decrease in the regions where *Cyp26* genes are expressed, and mice lacking either *Cyp26a1* or *Cyp26b1* die *in utero* or immediately after birth and exhibit abnormalities consistent with those seen in RA teratogenesis [18]–[20]. Thus, the coordinated regulation of the RALDH activity and the CYP26 activity determines the RA stimulation level.

In the present study, we detected the expression of *Cyp26b1* but not *Cyp26a1* or *Cyp26c1* in effector/memory populations of T cells from gut-related lymphoid organs but not those from skin-draining lymph nodes or the spleen. We also found that RA induced the expression of *Cyp26b1* but not *Cyp26a1* or *Cyp26c1* in T cells upon activation. Forced changes in the *Cyp26b1* expression affected the *Ccr9* expression. The RA-induced *Cyp26b1* expression was, however, markedly inhibited by TGF-β. The combination of RA and TGF-β induces the differentiation of CD4^+^ naïve T cells to Foxp3^+^ inducible regulatory T cells (iTreg) upon activation. Thus, CYP26B1 may not disturb RA signals in developing Foxp3^+^ iTreg.

## Results

### Expression of Cyp26b1 in CD4^+^ and CD8^+^ T cells in gut-related lymphoid organs

**Figure 1:**
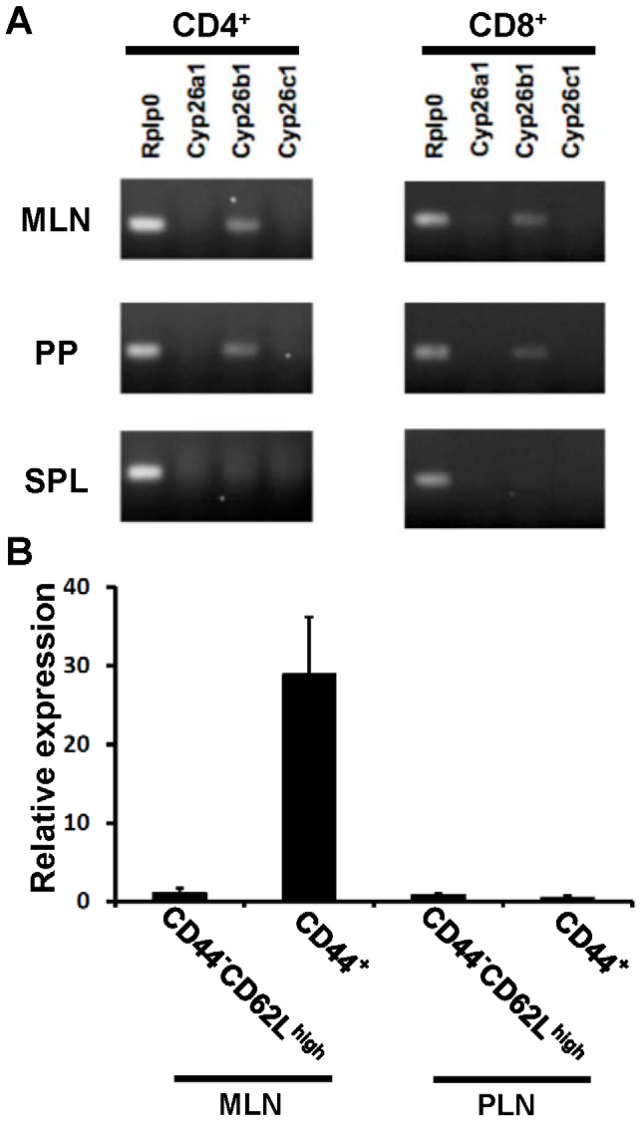
*Cyp26b1* is expressed in CD4^+^ and CD8^+^ T cells from gut-related lymphoid organs. (A) Expression of *Cyp26a1*, *Cyp26b1*, *Cyp26c1* and *Rplp0*, a loading control, in CD4^+^ or CD8^+^ T cells from MLN, Peyer's patches (PP) or spleens (SPL) was analyzed by RT-PCR. (B) Expression of *Cyp26b1* in CD4^+^CD44^−^CD62L^high^ or CD4^+^CD44^+^ T cells from MLN or peripheral lymph nodes (PLN) was analyzed by RT-PCR. Data are shown as the mean ± SD of triplicate cultures. Data are representative of three independent experiments.

CD4^+^ T cells and CD8^+^ T cells obtained from the gut-related lymphoid organs, mesenteric lymph nodes (MLN) and Payer's patches, expressed *Cyp26b1* but not *Cyp26a1* or *Cyp26c1* (Fig. 1A). Among CD4^+^ T cells in MLN, CD44^+^ effector/memory T cells but not CD44^-^CD62L^high^ naïve T cells expressed *Cyp26b1* (Fig. 1B). However, T cells from the spleen or skin-draining peripheral lymph nodes (PLN) did not significantly express any of the *Cyp26* genes (Fig. 1A, 1B and data not shown).

### RA induces Cyp26b1 expression in T cells upon activation

**Figure 2:**
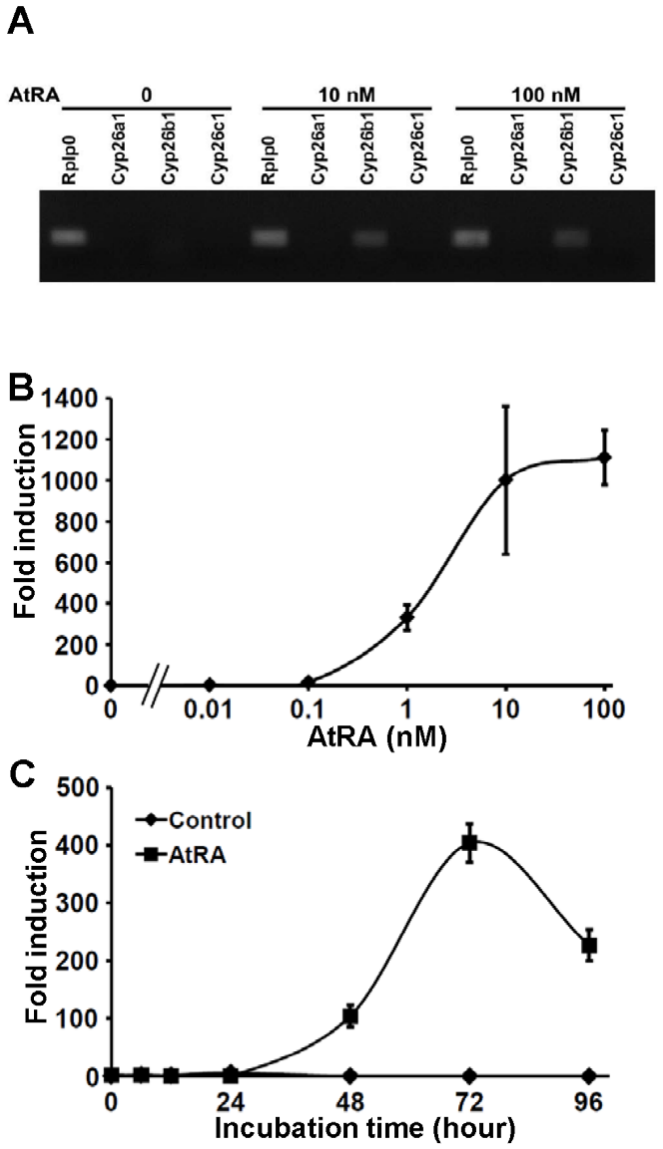
RA induces *Cyp26b1* expression in T cells. (A) Naïve CD4^+^ T cells were stimulated with antibodies to CD3 and CD28 in the presence or absence of AtRA (10 or 100 nM) for 2 days, and were further cultured with IL-2 and the same concentration without antibodies for 2 days. The cells were analyzed for the *Cyp26* expression by RT-PCR. *Rplp0* was used as a loading control. (B) Naïve CD4^+^ T cells were cultured as above but in the presence of graded concentrations of AtRA (0–100 nM). The mRNA levels were measured by quantitative real-time PCR. The expression levels are indicated as ‘fold induction’ relative to the level in the control cells without AtRA. (C) Naïve CD4^+^ T cells were cultured as above in the presence (squares) or absence (rhombuses) of 10 nM AtRA, and aliquots of the cells were harvested at 0, 6, 12, 24, 48, 72 and 96 h after the start of the culture. The mRNA levels were measured by quantitative real-time PCR. The expression levels are indicated as ‘fold induction’ relative to the level in the control cells without AtRA at 0 h. (B, C) Data are shown as the mean ± SD of triplicate cultures. Data are representative of three independent experiments.

As RA is consistently provided by DCs and some other cells in the gut-related lymphoid organs [1], [21], we examined if RA induced *Cyp26b1* expression in T cells. Indeed, the expression of *Cyp26b1* but not *Cyp26a1* or *Cyp26c1* was induced in naïve CD4^+^ T cells upon activation in the presence of the major physiologic RA, all-*trans*-RA (AtRA) (Fig. 2A). It has been previously demonstrated that the CYP enzymes CYP1A1, CYP3A and CYP2S1 also contributed to the 4-hydroxylation of AtRA [22], [23], but the expression of these enzymes was not detectable in either mock-treated or AtRA-treated T cells (data not shown). Even at a concentration as low as 1 nM, AtRA induced moderate but significant expression of *Cyp26b1* (Fig. 2B). In the presence of 10 nM AtRA, *Cyp26b1* expression became detectable after 48 h of stimulation of naïve CD4^+^ T cells with antibodies to CD3 and CD28. The cells were further cultured with AtRA and IL-2 but in the absence of the antibodies for another 48 h to induce CCR9 expression. The *Cyp26b1* expression reached a peak after 72 h and decreased subsequently (Fig. 2C).

### Inverse regulation of Ccr9 expression by altering Cyp26b1 expression

**Figure 3:**
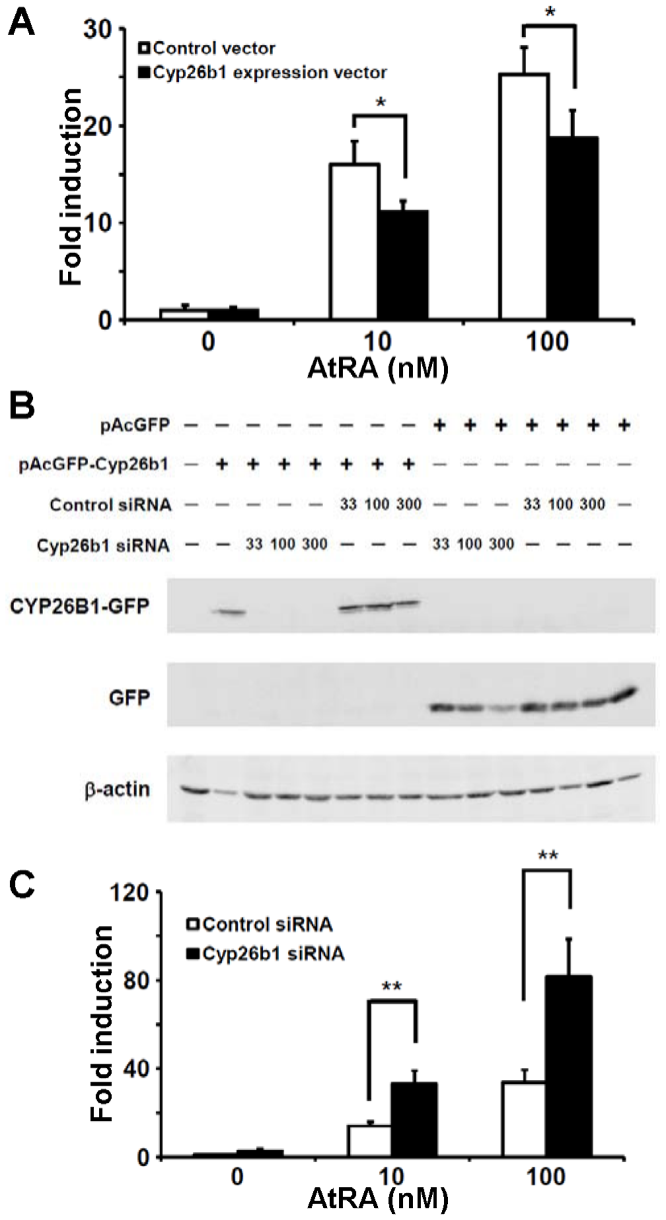
Inverse regulation of *Ccr9* expression by altering *Cyp26b1* expression. (A) Naïve CD4^+^ T cells were stimulated with antibodies to CD3 and CD28 for 2 days, and were transfected with the *Cyp26b1* expression vector or the corresponding insertless plasmid (Control vector). The cells were further stimulated with the antibodies for 24 h, and were transferred and cultured in new wells containing IL-2 but without antibodies. AtRA (10 or 100 nM) or vehicle control was added in all the cultures. The mRNA levels of *Ccr9* were measured by semi-quantitative real-time PCR. The expression levels are indicated as ‘fold induction’ relative to that in the control cells without AtRA. (B) COS7 cells were transiently transfected with the GFP expression vector (pAcGFP) or the CYP26B1-GFP fusion protein expression vector (pAcGFP-Cyp26b1) together with graded amounts of *Cyp26b1* siRNA or control siRNA (33, 100 or 300 nM). The cells were analyzed for the expression of CYP26B1-GFP protein and GFP protein by Western blotting with anti-GFP antibody. (C) Naive CD4^+^ T cells were stimulated for 2 days, and were transfected with 100 nM of *Cyp26b1* siRNA or control siRNA. After the culture, mRNA levels of *Ccr9* were measured by semi-quantitative real-time PCR. Data are expressed as ‘fold induction’ of the levels detected in the control siRNA transfected cells in the absence of AtRA. (A, C) Data are shown as the mean ± SD of triplicate cultures. Statistical significance was determined by the Student's *t*-test; **p < 0.01; *p < 0.05. Data are representative of three independent experiments.

We investigated whether *Ccr9* expression can be affected by an altered *Cyp26b1* expression in T cells. The *Ccr9* expression levels induced in T cells transfected with the pCMV5 expression vector containing the *Cyp26b1* cDNA (Cyp26b1 expression vector) were lower than those induced in T cells transfected with the pCMV5 vector alone (control vector) in the presence of 10 or 100 nM of AtRA (Fig. 3A). We also analyzed the effect of RNA interference-mediated silencing of *Cyp26b1*. In order to find out the optimal transfection conditions for an efficient reduction of CYP26B1 expression, we first transfected COS7 cells with either control short interfering RNA (siRNA) or *Cyp26b1* siRNA together with the expression vector encoding a CYP26B1-green fluorescent protein (GFP) fusion protein (pAcGFP-Cyp26b1), and analyzed their protein levels by Western blot using anti-GFP antibody. Compared with the control siRNA transfection, the *Cyp26b1* siRNA transfection dramatically reduced the CYP26B1-GFP expression levels (Fig. 3B). Depending on the results, we next transfected CD4^+^ T cells with 100 nM *Cyp26b1* siRNA, and assessed its effect on the *Ccr9* expression. Real-time PCR analysis revealed that AtRA-induced *Ccr9* expression levels were significantly higher in T cells treated with *Cyp26b1* siRNA than those treated with control siRNA (Fig. 3C). These results suggest that AtRA-induced *Ccr9* expression is inversely regulated by *Cyp26b1* expression.

### A CYP26 inhibitor upregulates RA-induced CCR9 expression

**Figure 4:**
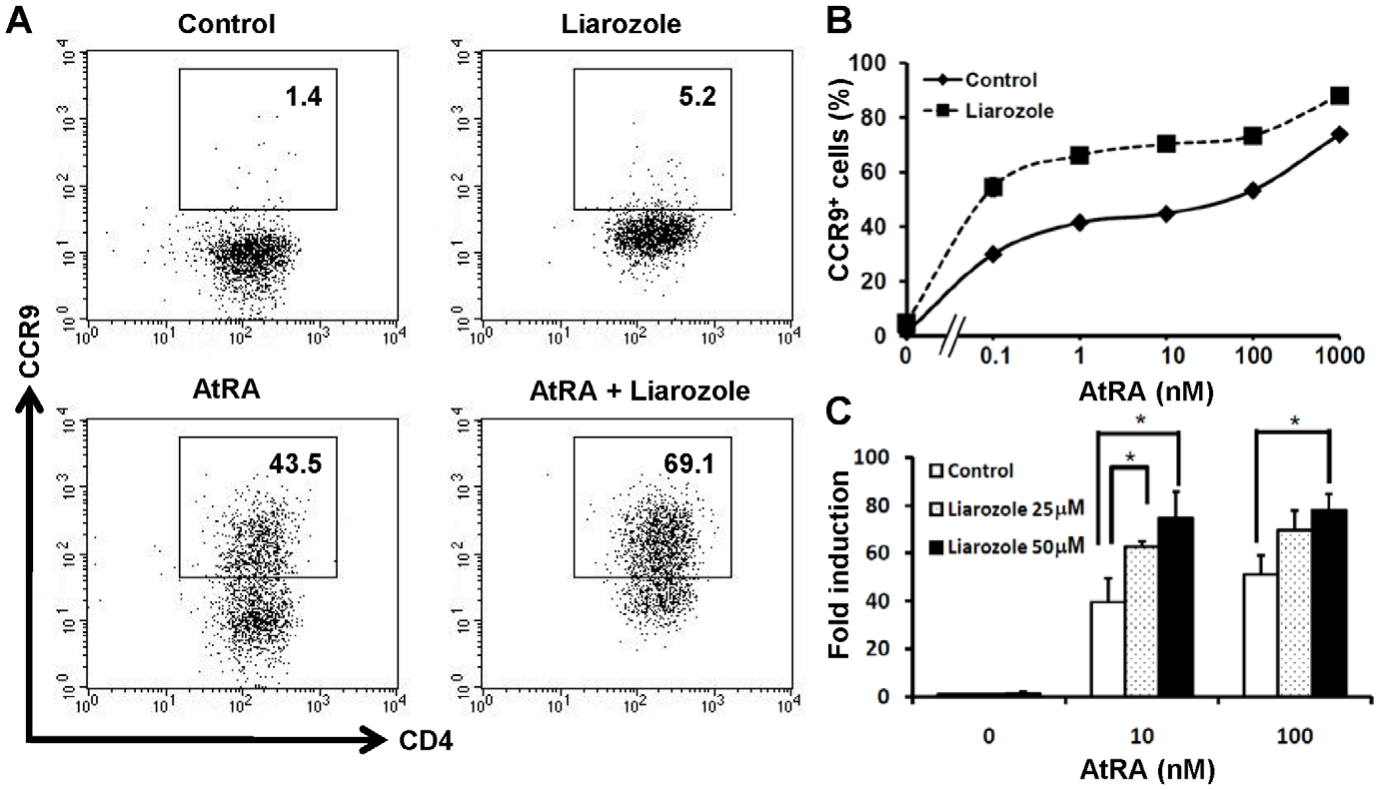
The CYP26 inibitor, Liarozole, upregulates RA-induced CCR9 expression. Naïve CD4^+^ T cells were cultured as described in the legend of Fig. 2A but in the presence or absence of Liarozole (50 µM) with or without AtRA (10 nM) (A) or with graded concentrations of AtRA (B). The cells were analyzed for the surface expression of CCR9 by flow cytometry. (C) Naïve CD4^+^ T cells were cultured as above in the presence or absence of Liarozole (25 or 50 µM) with or without AtRA (10 or 100 nM). *Ccr9* mRNA levels were measured by semi-quantitative real-time PCR assay. The expression levels are indicated as ‘fold induction’ relative to the level in the control cells without AtRA. (B, C) Data are shown as the mean ± SD of triplicate cultures. Statistical significance was determined by the Student's *t*-test; *p < 0.05. Data are representative of three independent experiments.

Liarozole is a standard inhibitor of CYP26 [24]. Liarozole significantly enhanced AtRA-induced CCR9 expression in T cells, although the inhibitor alone slightly upregulated the basal levels (Fig. 4A & 4B). The expression levels of *Ccr9* mRNA induced by 10 or 100 nM AtRA were also enhanced by Liarozole (Fig. 4C). The results indicate that CYP26B1 activity affects the AtRA-induced expression of CCR9 on T cells.

### TGF-β inhibits AtRA-induced Cyp26b1 expression

**Figure 5:**
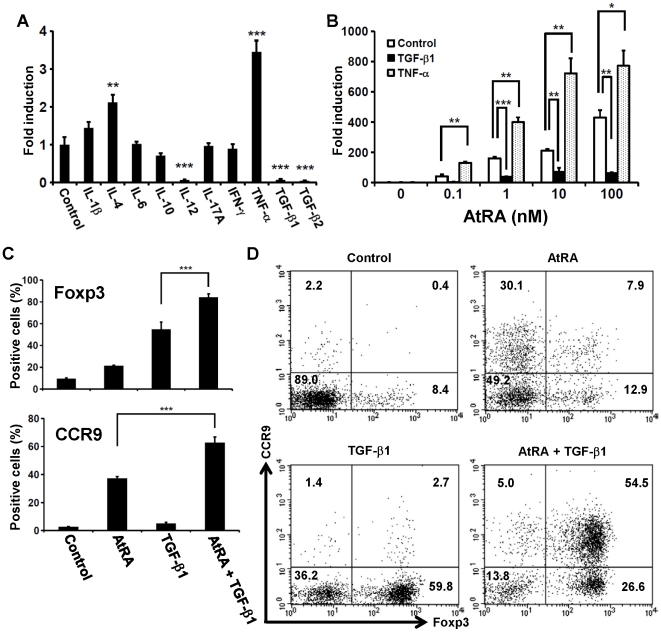
TGF-β inhibits RA-induced *Cyp26b1* expression. (A, B) Naïve CD4^+^ T cells were cultured as described in the legend of Fig. 2A but in the presence of various cytokines (10 ng/ml each) with AtRA (10 nM) (A), or graded concentrations of AtRA together with 4 ng/ml of TGF-β1 or 10 ng/ml of TNF-α (B). The expression of *Cyp26b1* was assessed by semi-quantitative real-time PCR. The expression level is indicated as ‘fold induction’ relative to the expression level in the AtRA-treated cells without cytokine (A), or the expression level in the control cells in the absence of AtRA (B). Data are shown as the mean ± SD of quadruplicate cultures (A) or triplicate cultures (B). Statistical significance was determined by the Student's *t*-test; ***p < 0.001; **p < 0.01; *p < 0.05, Cytokine-treated vs. Control for (*A*) or as indicated in the panel (*B*). (C, D) CD4^+^CD44^−^ T cells were cultured in the presence of AtRA (10 nM), TGF-β1 (4 ng/ml) or the combination of them for 4 days. The cells were analyzed for the expression of Foxp3 and CCR9 by flow cytometry. (C) Data are shown as the mean ± SD of triplicate cultures. Statistical significance was determined by the Student's *t*-test; ***p < 0.001. (D) Representative flow-cytometric profiles are shown. Data are representative of three independent experiments.

It is known that RA directly and indirectly modulates functional differentiation of naïve T cells [25]–[28]. As cytokines play critical roles in functional differentiation of naïve CD4^+^ T cells, we examined if cytokines might affect *Cyp26b1* expression in T cells upon activation. None of the cytokines we examined could induce *Cyp26b1* expression in the absence of AtRA (data not shown). However, TGF-β1, TGF-β2 and IL-12 dramatically inhibited the AtRA-induced *Cyp26b1* expression, whereas IL-4 and TNF-α significantly enhanced the expression (Fig. 5A & 5B). On the other hand, IL-1β, IL-6, IL-10, IL-17A or IFN-γ was of little effect on the expression. It is known that the combination of RA and TGF-β efficiently induces differentiation of naïve CD4^+^ T cells into Foxp3^+^ iTreg [6]–[11]. Thus, during the differentiation into Foxp3^+^ iTreg, TGF-β might protect RA-dependent signals by inhibiting the CYP26B1 expression. In fact, TGF-β significantly enhanced the RA-induced CCR9 expression on T cells (Fig. 5C & 5D). Meanwhile, RA significantly enhanced Foxp3 expression on T cells as reported [6]–[11].

## Discussion

In the present study, we found that *Cyp26b1* is expressed in antigen-experienced T cells from gut-related lymphoid organs, and that RA induced *Cyp26b1* expression in naïve T cells upon activation (Fig. 1 & 2). Expression of the other *Cyp26* family members were not detected in T cells (Fig. 2A), although, in other cell types, their expression can be also induced by RA [29]–[31]. The *Cyp26b1* expression in T cells became detectable after 48 h of culture in the presence of 10 nM AtRA (Fig. 2C). Previous studies, however, showed that *Cyp26a1* mRNA expression was induced within 2 h of culture with AtRA in human keratinocyte-derived HaCaT cells and within 3 h in NB4 and HL-60 cells [31], [32]. These results suggest that the expression of *Cyp26a1* is far more rapidly induced than that of *Cyp26b1*. In the upstream region of the *Cyp26a1* gene in human and mouse, there are two typical retinoic acid-response elements (RAREs) and a G-rich element. These motifs appear to function synergistically to provide maximal induction of *Cyp26a1* in response to AtRA [33], [34]. By contrast, little is known about the regulatory mechanisms of the *Cyp26b1* gene expression, and thus far no functional RARE has been reported. More than 500 genes have been suggested to be regulatory targets of AtRA, and approx. 75% of these genes are likely to be regulated indirectly [35], [36]. Considering the lack of an apparent RARE in the promoter region of the *Cyp26b1* gene and its delayed expression, the expression of *Cyp26b1* is likely to be regulated indirectly by RA.

It was reported that constitutive expression of *Cyp26a1* cDNA in P19 and HeLa cells made them hyposensitive to RA [15]. On the contrary, the CYP26 inhibitor ketoconazole enhanced AtRA-dependent reporter gene expression in HaCaT cells [32]. CYP26A1 and CYP26B1 exhibit a high degree of specificity for AtRA, while CYP26C1 can catabolize both AtRA and 9-*cis*-RA [30], [37], [38]. Therefore, the intracellular availability of AtRA and the AtRA-dependent gene expression can be controlled by the expression and activity of CYP26 enzymes. Our present results suggest that AtRA-induced *Ccr9* expression in T cells is influenced by the expression and activity of CYP26B1 (Fig. 3 & 4). CYP26B1 may thus affect the tissue homing specificity of T cells.

IL-4 and IL-12 affected the AtRA-induced expression of *Cyp26b1* (Fig. 5A). We have previously shown that AtRA directly suppresses IL-12-dependent differentiation of naïve CD4^+^ T cells to Th1 cells, and enhances or suppresses IL-4-dependent differentiation to Th2 cells depending on its concentration and the timing of its addition [27]. Thus, CYP26B1 may not disturb the RA-dependent suppression of Th1 differentiation, but may modulate the RA effect on Th2 differentiation in the RA-rich microenvironment like in the small intestinal tissue. We also found that TGF-β markedly inhibited AtRA-induced expression of *Cyp26b1* (Fig. 5A & 5B). It has long been known that cross-talk between RA and TGF-β is crucial in embryonic development. Retinoid imbalance affects TGF-β expression in embryo, and causes teratogenic effects [39], [40]. TGF-β is known to suppress the expression of RA receptors and a cytoplasmic RA-binding protein in embryonic cells [41], [42]. Cross-talk between RA and TGF-β also plays important roles in functional differentiation of T cells. We found that TGF-β significantly enhanced AtRA-induced CCR9 expression (Fig. 5C & D). On the other hand, AtRA enhanced TGF-β-induced Foxp3 expression on T cells as previously reported [6]–[11] (Fig. 5C & D). RA signals that enhance TGF-β-dependent differentiation of Foxp3^+^ iTreg and their acquisition of gut-homing specificity were unlikely to be suppressed by CYP26B1. Aberrant expression of CYP26B1 may disturb not only the tissue homing specificity but also the differentiation into iTreg. On the other hand, TNF-α enhanced RA-induced expression of *Cyp26b1* (Fig. 5B). As TNF-α contributes to inflammatory responses through diverse biological actions, T cells activated in the presence of TNF-α may actively diminish RA signals that induce immunosuppression and/or homing into uninflamed gut tissues.

Inflammatory bowel diseases (IBD) represent chronic relapsing and remitting inflammatory disorders of the gastrointestinal tract, and are characterized by leukocytic infiltration of the intestinal mucosa. In peripheral blood lymphocytes, CCR9-positive T cells were markedly elevated in patients with Crohn's disease [43]. It was also shown that the percentage of CCR9-positive lymphocytes increased in murine model of Crohn's disease, and neutralization of the receptor or the chemokine attenuated early disease [44]. These CCR9-positive lymphocytes might be mainly pro-inflammatory but not anti-inflammatory, as the balance between regulatory T cells and Th17 may affect the disease outcome [45]. It is becoming likely that RA-signal levels in the gut largely affect the IBD development. The regulation of retinoid metabolism may offer novel therapeutic strategies for treatment of IBD.

## Materials and Methods

### Ethics statement

All animal experiments were performed according to the protocols approved by the Animal Care and Use Committee of Tokushima Bunri University (protocol # KP09-41-001).

### Mice

B10.D2 mice and TCR-DO11.10/Rag2^−/−^ (B10.D2/AiTac-TgN(DO11.10)-*Rag2* ^tm1^) mice were from Japan SLC (Hamamatsu, Japan) and Taconic Farm (Hudson, USA), respectively. All animals were maintained in the specific pathogen free condition in the animal facility of Tokushima Bunri University at Kagawa Campus.

### Isolation of lymphocytes from secondary lymphoid tissues

Single cell suspensions were prepared from MLN, Peyer's patches, PLN and spleens of B10.D2 mice. For isolation of CD4^+^ T cells, CD4^+^ T cells were positively isolated using Dynabeads® Mouse CD4 (Invitrogen, Carlsbad, USA). For isolation of CD8^+^ T cells, CD8^+^ T cells were stained with phycoerythrin (PE)-conjugated anti-CD8α antibody (BD Biosciences, Franklin Lakes, USA) and positively isolated using anti-PE antibody-coupled magnetic cell-sorting microbeads (Miltenyi Biotec, Bergisch Gladbach, Germany). Purified CD4^+^ and CD8^+^ T cells were immediately used for the RNA extraction. Naïve CD4^+^CD62L^high^ T cells were purified as previously described [1], [46]. For sorting of effector/memory CD4^+^ T cells, cells were purified using Easysep® mouse CD4^+^ T cell enrichment kit (Stemcell, Vancouver, Canada), and were stained with PE-Cy7^™^-conjugated anti-CD4 (BioLegend, San Diego, USA), fluorescein isothiocyanate (FITC)-conjugated anti-CD62L (BD Biosciences) and allophycocyanin (APC)-conjugated anti-CD44 (BioLegend) antibodies. CD4^+^CD44^+^ and CD4^+^CD44^−^CD62L^high^ cells were sorted on a BD FACSAria^™^ (BD Biosciences), to >95% purity.

### Naïve T cell culture

Naïve CD4^+^ T cells were cultured as previously described with a slight modification [1], [46]. Briefly, the cells were added into the plates coated with 5 µg/ml of anti-CD3 (145-3C11) and 1 µg/ml of anti-CD28 (BioLegend) antibodies, and cultured for 2 days in the presence of AtRA. The cells were then transferred into the medium containing 20 units/ml of IL-2 and AtRA, and cultured for another 2 days. In some experiments, cytokines (Peprotech, Rocky Hill, USA) or Liarozole (Tocris Bioscience, Ellisville, USA) was added to the culture. For the induction of Foxp3^+^ T cells, CD4^+^CD44^−^ T cells from spleen and MLN of B10.D2 mice were isolated by negative selection using an EasySep® Mouse CD4^+^ T cell enrichment kit supplemented with biotinylated anti-CD44 antibody (BioLegend). The cells were added into the plates coated with 5 µg/ml of anti-CD3 and 1 µg/ml of anti-CD28 antibodies, and cultured for 2 days in the presence of 10 nM of AtRA, 4 ng/ml of TGF-β1 or the combination of the two. The cells were then transferred into the medium containing 1.25 units/ml of IL-2 with or without AtRA and TGF-β1, and cultured for another 2 days.

### Transfection of COS7 cells

The cDNA sequence of *Cyp26b1* containing the entire open reading frame was amplified by PCR and cloned into pAcGFP1-N1 (BD Biosciences). The siRNA cocktail targeting the mouse *Cyp26b1* was purchased from B-Bridge international Inc. (Cupertino, USA), which contains 3 siRNAs (Table S1). The control siRNA cocktail was also purchased from B-Bridge International Inc. (catalog number S6C-0126). COS7 cells were transfected with the expression vector with or without the cDNA together with siTrio *Cyp26b1* siRNA or siTrio negative control siRNA using Lipofectamine™ 2000 reagent (Invitrogen). The transfected cells were lysed at 48 h post-transfection.

### Immunoblot analysis

A.v. Peptide Antibody (Clontech, Mountain View, USA) was used to detect GFP-fused proteins. Cells were lysed for 30 min on ice in 1% (v/v) Triton X-100, 150 mM NaCl, 20 mM Tris-HCl, 1 mM EDTA and 1% (v/v) protease inhibitor cocktail (Nacalai tesque, Kyoto, Japan), and cell lysates were centrifuged at 20,000×*g* for 5 min. Proteins in cell lysates were separated by SDS-PAGE, and were transferred to nitrocellulose membranes (GE Healthcare, Little Chalfont, UK). The membranes were probed with A.v. Peptide Antibody followed by incubation with horseradish peroxidase-conjugated goat anti-rabbit IgG Ab (Zymed Laboratories, South San Francisco, USA) and were developed by ECL^™^ Western Blotting Detection Reagents (GE Healthcare), and bands were visualized by a LAS-3000 (Fujifilm, Tokyo, Japan).

### Transfection of CD4^+^ T cells

Transfection of CD4^+^ T cells was performed by electroporation using Mouse T Cell Nucleofector Kit (Amaxa, Gaithersburg, USA). Naïve CD4^+^ T cells were stimulated with plate-bound antibodies to CD3 and CD28 as described above. After 2 days of stimulation, T cells (1×10^6^ cells) were transfected with 4 µg of the *Cyp26b1* expression vector (pCMV5-Cyp26b1) or the corresponding insertless plasmid (pCMV5). After electroporation, the cells were re-stimulated for 1 day with plate-bound antibodies to CD3 and CD28. The cells were then transferred into the medium containing 20 units/ml of IL-2 with or without AtRA, and cultured for another 2 days. For the siRNA knocking down study, naïve CD4^+^ T cells were stimulated with anti-CD3 and anti-CD28 antibodies coated on the plates described as above. After 2 days of stimulation, T cells (1×10^6^ cells) were transfected with 100 nM of siTrio *Cyp26b1* siRNA or siTrio negative control cocktail. After electroporation, the cells were re-stimulated for 1 day with or without AtRA with anti-CD3 and anti-CD28 antibodies coated on the plates as described above. The cells were then expanded in the medium containing 20 units/ml of IL-2 with or without AtRA for another 2 days.

### Real-time PCR

Real-time PCR was performed as previously described with a slight modification [46]. Total RNA was isolated from cells using RNeasy® Mini Kit (Qiagen, Hilden, Germany), and cDNA was generated using QuantiTect® Reverse Transcription Kit (Qiagen). cDNA was used as a template for real-time PCR with Power SYBR® Green Master Mix (Applied Biosystems, Foster City, USA) and gene-specific primers (Table S2). The reaction was performed with Applied Biosystems 7500 real time PCR system (Applied Biosystems). Analysis was performed with Applied Biosystems 7500 real time PCR system or fractionation in a 2% (w/v) agarose/TAE gel. Real-time PCR conditions consisted of an initial denaturing step at 50°C for 2 min and 95°C for 10 min, followed by 40 cycles of denaturation for 15 s at 95°C, annealing and extension for 1 min at 60°C. All reactions were performed in triplicates.

### Fluorescence-activated cell-sorting (FACS) analysis

The cells were stained with APC-conjugated anti-CD4 (BioLegend) and PE-conjugated anti-CCR9 (R&D Systems, Minneapolis, USA) antibodies in the presence of anti-FcR antibody, and were analyzed with a BD FACSCalibur^™^ instrument and the BD CellQuest™ Pro software (BD Biosciences). Intracellular staining of Foxp3 was performed using APC mouse/rat Foxp3 staining set (eBioscience, San Diego, USA).

## Supporting Information

Table S1 **Sequences of siRNAs using knocking down the** ***Cyp26b1*** **expression.**
(DOC)

Table S2 **Sequences of gene specific primers for RT-PCR.**
(DOC)
