# Supplementary material for: Cyp26b1 Regulates Retinoic Acid-Dependent Signals in T Cells and Its Expression Is Inhibited by Transforming Growth Factor-β
Source: PLoS One. 2011 Jan 7;6(1):e16089. doi: 10.1371/journal.pone.0016089 (PMC3017564; doi:10.1371/journal.pone.0016089)
Supplement: Table S1 — Sequences of siRNAs using knocking down the Cyp26b1 expression. (DOC) [file pone.0016089.s001.doc]

**Table S1**: Sequences of siRNAs using knocking down the *Cyp26b1* expression.

SMF27A-1536-1 5′-GUG CGG AGA AUG UGC GCA ATT-3′ (sense)

5′-UUG CGC ACA UUC UCC GCA CTT-3′ (antisense)

SMF27A-1536-2 5′-CCU UCG AAC UGG AUG GUU UTT-3′ (sense)

5′-AAA CCA UCC AGU UCG AAG GTT-3′ (antisense)

SMF27A-1536-3 5′-GUU CUU UGG UAG AGG GUU ATT-3′ (sense)

5′-UAA CCC UCU ACC AAA GAA CTT-3′ (antisense).
