# Supplementary material for: Cyp26b1 Regulates Retinoic Acid-Dependent Signals in T Cells and Its Expression Is Inhibited by Transforming Growth Factor-β
Source: PLoS One. 2011 Jan 7;6(1):e16089. doi: 10.1371/journal.pone.0016089 (PMC3017564; doi:10.1371/journal.pone.0016089)
Supplement: Table S2 — Sequences of gene specific primers for RT-PCR. (DOC) [file pone.0016089.s002.doc]

**Table S2**: Sequences of gene specific primers for RT-PCR.

*Cyp26a1*-specific primers 5′-CGA TTG AAT CCT CCG GTC C-3′ (forward)

5′-CCA CAT CGT GGG TGT CAC AG-3′ (reverse)

*Cyp26b1*-specific primers 5′-AGA GCA GCA AGG AAC ATG GC-3′ (forward)

5′-AGT TGC ATG ATC AAG GAT GTG C-3′ (reverse)

*Cyp26c1*-specific primers 5′-AGG TGA TGC CCT GCT CTT GAT-3′ (forward)

5′-GCT GGC TGT GGT GAA AAA GG-3′ (reverse)

*Ccr9*-specific primers 5′-TGC CAT GTT CAT CTC CAA CTG-3′ (forward)

5′-GAA CTG GGT TCA GAC AAC TGT GG-3′ (reverse)

*Rplp0*-specific primers 5′-GGT GCC ACA CTC CAT CAT CA-3′ (forward)

5′-CGC AAA TGC AGA TGG ATC AG-3′ (reverse)

*Cyp1a1*-specific primers 5′-GCT CCT GGC TGT CAC CGT AT-3′ (forward)

5′-CAT GTG ACC AAT GAA GGG CA-3′ (reverse)

*Cyp3a11* (the orthologue of human *Cyp3a*)-specific primers

5′-TCA CTG GAA ACC TGG GTG CT-3′ (forward)

5′-CTA AAA ATG GCA GAG GTT TGG G-3′ (reverse)

*Cyp2S1*-specific primers 5′-GGC TTC TTT TTG GAG AGG GC-3′ (forward)

5′-GGC GCA TAC CTG TAA TTC CAG-3′ (reverse)
